# Enhancing Product Quality in High-Variant Manufacturing: Combining Physics-Based Simulations and Data Science for Target Variable Estimation in an IoT- and Machine Learning-Driven Context

**DOI:** 10.3390/s26030830

**Published:** 2026-01-27

**Authors:** Manuela Larissa Schreyer, Alexander Gerber, Steffen Neubert, Peter Simon

**Affiliations:** 1 Austria Metall GmbH, Lamprechtshausenerstr. 61, 5282 Braunau am Inn, Austria; 2 AMAG Casting GmbH, Lamprechtshausenerstr. 61, 5282 Braunau am Inn, Austria; 3 AMAG Rolling GmbH, Lamprechtshausenerstr. 61, 5282 Braunau am Inn, Austria

**Keywords:** process optimization, machine learning, heterogeneous production processes, data science, target variables, limited dataset

## Abstract

Due to growing demands for quality, sustainability, and digitalization, data science and artificial intelligence are gaining importance across industries. The extensive product range in many sectors often poses considerable challenges. For example, machine learning (ML) models may struggle with limited data per production variant. The present paper proposes a methodology that integrates the fields of data science and physical simulations. The results from finite element method (FEM) simulations are utilized to transform the process data in such a manner that it can be compared across processes for different production variants and employed for machine learning (ML) methods and statistical analyses. The method is illustrated using an example of aluminum production. A key advantage of this approach is that it can effectively model even production variants with very low quantities. The following discussion will present how this method can be used to enhance production processes, specifically to identify parameters that directly influence product quality, which would not be evident using alternative approaches. Furthermore, the work explores the potential for precisely controlling these parameters using ML models and discusses some major challenges.

## 1. Introduction

The fields of data science and artificial intelligence are assuming an increasingly important role in a multitude of industries. This is primarily attributable to an increased demand for higher quality and sustainability, in conjunction with the objective of enhancing efficiency and competitiveness [1,2]. The capacity to analyze large amounts of data enables automated and more precise decision-making. Furthermore, advancements in digitalization are instrumental in facilitating the integration of data-driven models.

Data-driven processes enable the efficient and precise handling of a wide variety of issues. The present article focuses on the optimization of production processes, with a particular emphasis on reducing waste and improving product quality. This paper analyses a selection of key challenges that can arise in the field of data science, as observed in many industrial sectors. It demonstrates possible solutions in the context of a specific use case in the aluminum processing industry, with a particular focus on aluminum ingot casting at AMAG.

The large quantity and complexity of production data necessitate the implementation of efficient techniques to derive insights that may facilitate the further optimization of production processes. In [3], for instance, a seven-step problem-solving strategy for the sustainable implementation of big data projects is presented. As is the case in numerous industrial sectors, the product range is particularly extensive. In industry, this means that many different product variants are manufactured. For instance, the manufacturing of products of various sizes or material compositions. Therefore, the data is very heterogeneous, and some product variants are produced only rarely, such as custom-made products. Conversely, other product variants are manufactured in large quantities. However, a model should be capable of covering all cases equally well, including individual cases with low production volumes, as these are often associated with higher margins. This and other factors can result in significant challenges when conducting statistical analysis and building machine learning models [3].

In many cases, a substantial database is available for use in this context. However, the individual samples in the database are frequently not comparable due to the extensive product range and the resulting very different production histories. The wide range of products and the large variety of production settings pose a considerable challenge for statistical analyses and machine learning due to the extremely heterogeneous data, which leads to poor model generalization [4,5]. Conventional models produce distorted or inaccurate results due to this variability, as they do not consider the differences between settings [4]. There are various approaches to overcoming this challenge, such as clustering or data transformation methods, which help to extend analysis and models to infrequently produced variants [6].

**Figure 1:**
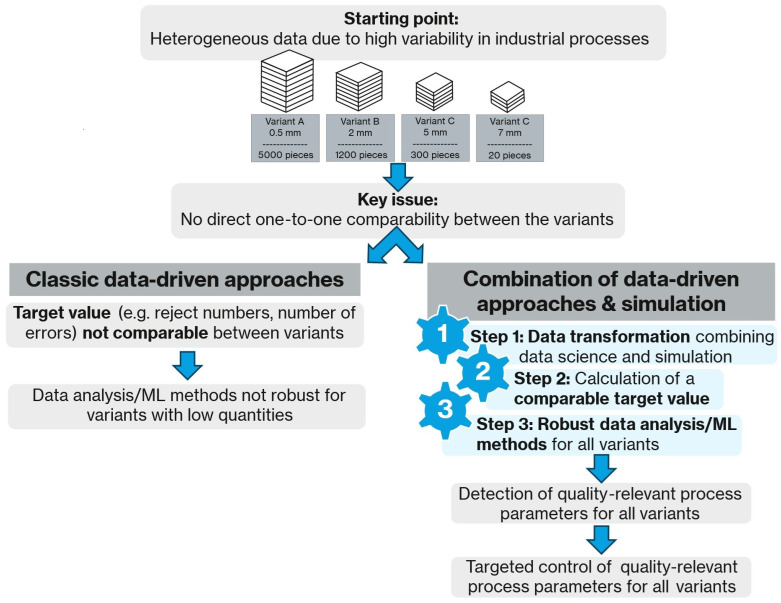
Schematic representation of the research framework.

Figure 1 illustrates the objective and research framework of this article. The issue under discussion is rooted in the presence of heterogeneous data, a consequence of the high variability inherent in industrial processes, as can be observed in the domains of metal or component manufacturing, just to name two examples. A plant could produce similar products, with slight variations, such as different geometries or customer specifications. For illustrative purposes, Figure 1 presents metal sheets of varying thicknesses.

As illustrated on the left-hand side of the figure, conventional data-driven methods frequently encounter limitations when confronted with a substantial number of product variants and a limited number of samples from certain variants. As the target variable (e.g., reject numbers, number of defects) is frequently non-comparable between different variants, data analysis and machine learning methods can only be applied per product variant. This means that data analysis/machine learning (ML) methods are not robust for variants with low quantities.

Referring to the right-hand side of Figure 1, the combination of data-driven approaches and physical simulation is demonstrated. The integration of physics-based simulations, particularly the finite element method (FEM), with data science methodologies enables mathematical modelling and neutralization of the effects of individual production steps. The employment of this data transformation method results in the generation of a robust and comparable target variable that reliably captures even rare product variants, serves as a quality indicator, and establishes the foundation for effective statistical analyses and machine learning models. The fundamental concept underlying this approach involves a form of cross-process normalization or standardization, grounded in physical understanding derived from the FEM simulations. This enables the representation of diverse production conditions through a common target variable. These transformed target variables can then be used for statistical analyses or machine learning methods.

The method has been developed for the purpose of enabling the identification and control of quality-relevant process parameters. This is achieved even in cases where the data sets are heterogeneous, and the sample size is small for certain product variants. As demonstrated in this article, the practical implementation and validation of the methodology in an industrial environment has been shown to result in a reduction in waste, an enhancement of process stability, and an improvement in efficiency.

In summary, the integration of data science and physical simulation can be used for reliable calculation of target variables. These target variables, in turn, form the basis for data-driven optimization of product quality (i.e., the detection and control of quality-relevant process parameters) in complex, highly varied manufacturing processes. These three aspects are therefore not independent of each other, but rather build directly on each other methodologically.

The key innovative contributions of this article are as follows:

- The paper presents the development and application of a combined method of physical simulation and data-driven techniques. This method uses simulation results to transform real data through mathematical modelling and neutralising the effects of individual production steps to generate a target value that can be compared across different process variants. This target value is suitable for ML methods and statistical analyses.
- The methodology is demonstrated as a means of mathematically neutralizing ‘hidden correlations’ and process-related influences, including those caused by downstream production steps. The purpose of this is to identify the actual causes of quality deviations.
- The practical implementation and validation of the proposed method is illustrated through the case study of aluminum ingot production.
- The transferability of the approach to other industrial contexts characterized by high variant diversity and low quantities is discussed. In addition, the economic benefits of the approach are presented, including the significant reduction in scrap and the improvement of process stability.

The present article proposes a novel methodology that demonstrates the efficacy of integrating simulation and data science to calculate a comparable target variable in complex production processes for data analysis and machine learning methods. This facilitates process-data-based analysis of factors influencing product quality, even under difficult industrial conditions. This work makes a significant contribution to the future viability of data-driven production in Industry 4.0.

Article [7] provides a systematic overview of the current state of research on predictive quality in the manufacturing industry and summarizes more than 100 articles on this topic. The article also refers to simulations in the context of predictive quality and machine learning. Simulations are utilized for the purpose of generating virtual training data, which is then employed in the development of ML models. Moreover, classic simulations can be replaced by deep learning-based surrogate models, or individual components of classic simulation chains can be improved by deep learning. The articles [8,9] provide a comprehensive overview of the current state of the art and the most important methods in this field.

The concept delineated in this article integrates seamlessly into the framework of predictive quality and data-driven manufacturing, as it facilitates a combination between physical-based simulations and data-driven methods. The innovative aspect of this research lies in the utilization of cross-process normalization of real data using simulation results, with the purpose of making different production data comparable and usable for ML models—even in complex, highly variable processes. This creates a reliable data basis for a more precise analysis of the influence factor and for production control.

The article is divided into two main chapters, which describe two different steps. In the first step (Section 2: Methodological Framework—Calculating a Target Value), the target variable is first calculated using a combination of mathematical–statistical methods and physics-based simulations (e.g., FEM). The objective is to neutralize hidden correlations and influences from multi-stage processes, such as the influence of the rolling process on the scrap rate. This results in a target variable that is independent of downstream process steps and thus enables comparability between different product variants.

In the second step (Section 3: Results—Analysis of Influencing Factors on the Target Variable and ML-Based Parameter Control), the calculated target variable is used as the basis for the analysis of influencing parameters. The target variable calculated in Section 2 offers a clear advantage in that it enables reliable detection of correlations and identification of the most important quality-relevant process parameters, even when dealing with small or unbalanced data sets (e.g., rarely produced variants). The practical benefits of this are then demonstrated through the targeted controlling of influencing parameters using ML models.

The examples in each section offer practical illustrations of the theoretical approaches, demonstrating how the methods are applied in an industrial environment. The examples are embedded in the respective sections to illustrate the methodological step-by-step process. The recurring sections ‘General Approach’ provide an abstraction of the findings and demonstrate how the methods described can be transferred to other processes or industries. This makes the practical applicability and transferability of the methodology clear and comprehensible.

In more detail, Section 2 introduces the methodology of combining physics-based FEM simulations with data science approaches. The objective of this combination is to define a robust and comparable target variable for the implementation of effective statistical analyses and ML models in complex, multi-stage production processes characterized by a high degree of variability. Section 2 provides a detailed explanation of the methodological framework. Utilizing a real-world application from aluminum production, Section 2.1 demonstrates that a significant advantage of this method is that production variants, even those in very small quantities, can be effectively modelled and employed for data analysis and machine learning methods. The challenges associated with multi-stage production processes are discussed in Section 2.2. The present paper elucidates the circumstances in which conventional methodologies reach their limits and the manner in which a comparable target variable can be created by combining FEM simulations and mathematical transformation. The subsequent section (Section 2.3) provides a detailed exposition of the practical implementation and validation of this method in an industrial environment.

The present study compares data analyses based on the transformation method presented with non-transformed data, as demonstrated in Section 3. The purpose of this comparison is to demonstrate the effectiveness of the implemented method. The objective is to illustrate the identification of process parameters through data analysis, which exert a substantial impact on product quality and would have remained unnoticed without the implementation of the method.

Section 3.1 provides a detailed exposition of the calculation of the target value throughout the process chain for the specified use case. This is followed in Section 3.2 by an analysis of a critical process signal, the influence of which on product quality can be made visible using the new methodology. Section 3.3 elucidates the manner in which the insights obtained—particularly those pertaining to parameters that exert a direct influence on product quality—contribute to enhanced production efficiency. Furthermore, the potential for precise control of these parameters through the utilization of machine learning methods, along with the associated requirements and challenges, is assessed.

The manuscript concludes with a discussion and conclusions, which are presented in Section 4 and Section 5. The Discussion critically evaluates the results, addresses limitations and open questions, and assesses the transferability to other industrial contexts. The Conclusion summarizes the most important findings and provides an outlook on future research and application possibilities.

## 2. Methodological Framework—Calculating a Target Value

### 2.1. Use Case: Application Example and Description of the Data

**Figure 2:**
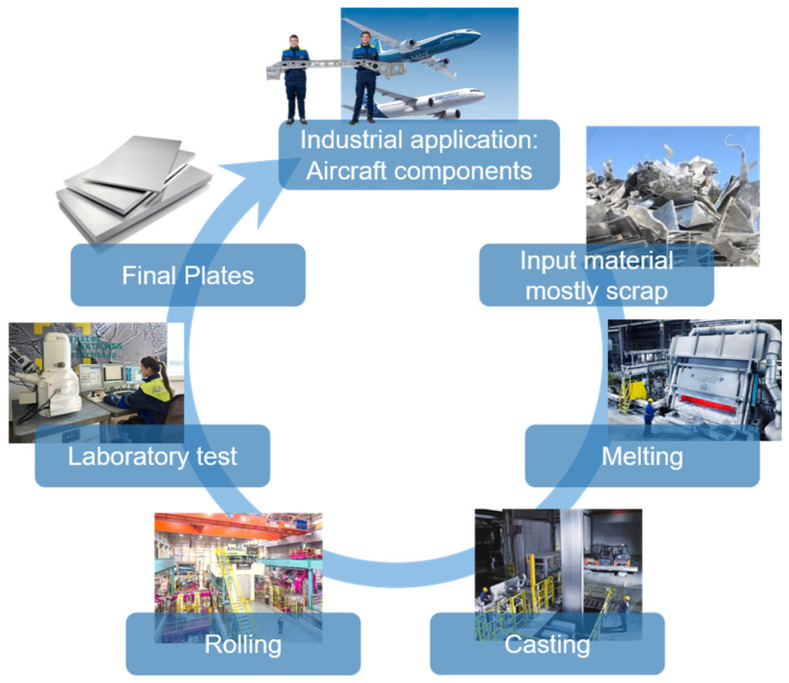
Schematic representation of the production process.

This application example deals with the production of plates for the aerospace industry, in particular with the casting of aluminum ingots, which are then subsequently rolled into plates from which aerospace components are finally milled. A schematic representation of the production process is shown in Figure 2. The production process begins with the input material, which consists mainly of recycled aluminum scrap, supplemented by primary aluminium from electrolysis. Before the material enters the smelting furnaces, it is carefully sampled to identify the alloy composition and any possible impurities such as plastics or foreign metals. The material is then processed in modern, energy-efficient smelting furnaces and subjected to chemical analysis to precisely adjust the desired alloy.

The liquid aluminium is then cast into rolling ingots. Among other things, the electromagnetic casting process is used here to ensure the particularly high quality of the ingots. These ingots are turned into plates or coils in a multi-stage rolling process. This process includes homogenisation, hot and cold rolling, heat treatment, stretching, and cutting. During these steps, numerous sensors continuously monitor parameters such as temperature, rolling pressure, and speed to ensure maximum precision.

Before the products are delivered, they undergo extensive laboratory and quality testing. This includes ultrasonic testing to detect non-metallic inclusions, as well as mechanical and chemical analyses to ensure that all customer specifications are met. Following the completion of rigorous testing procedures, the final plate is carefully packaged and delivered to the customer. The end products are used in industries such as aviation, automotive, packaging, and construction, where they are further processed and integrated into high-quality applications.

During the casting of the ingots, it is possible that non-metallic inclusions may be formed in the material, either as a result of the melting and casting process itself or as a consequence of the used input material. Such non-metallic inclusions may result in the rejection of the final product. This is determined during laboratory testing after the ingots have been rolled into plates [2,6,10]. Depending on the intended use of the end product, the final product must fulfil strict quality requirements, which are ensured by appropriate quality controls [11]. Among other things, the plates are subjected to ultrasonic (US) testing to detect non-metallic inclusions. If a plate contains too many or too large indications, the entire plate is rejected.

It is important to note that the origin of the non-metallic inclusions can be attributed to the casting process. But for technical reasons, the US test can only be carried out on the final plates, rather than immediately after the casting process on the cast ingot. Nevertheless, this implies that numerous production processes (e.g., homogenisation, rolling, heat treatment, stretching, cutting, etc.) occur between the initial creation and subsequent detection, which can span several weeks [2].

The primary aim is to reduce the rejection rate (percentage of products that are rejected due to quality issues), the plant utilization rate and, consequently, the delivery time. One approach to achieving this is to identify potential influencing factors in the formation of non-metallic inclusions during the casting process using statistical analysis and machine learning models. Some crucial considerations that must be addressed are highlighted in Section 2.2.1, Section 2.2.2 and Section 3.1.

As will be demonstrated in the following paragraphs, this example serves also to illustrate the significant impact which the rolling production process can have on the rejection rate with respect to casting-related non-metallic inclusions. This, in turn, has implications for statistical analyses and machine learning models, as already mentioned. Subsequently, methods from statistics are presented that can take this aspect into account in order to generate a target variable for statistical analyses and machine learning models that is independent of the subsequent production process. The issue of multi-step production manifests itself in a variety of industrial sectors. In the field of statistics, there exists a multitude of methodologies for the calculation of a target variable that is independent of ‘intermediate steps’ [6,12,13]. The present paper proposes one such methodology and discusses its advantages over alternative approaches.

In the field of statistics, the term “target variable” is used to describe the variable whose prediction or explanation is being sought as part of an analysis or prediction model. It represents the result or answers that can be predicted by analyzing the relationship to other explanatory variables (predictors). In this application example, the target variable should describe the non-metallic inclusions created by the casting process (in terms of size and number), regardless of all subsequent processes.

**Figure 3:**
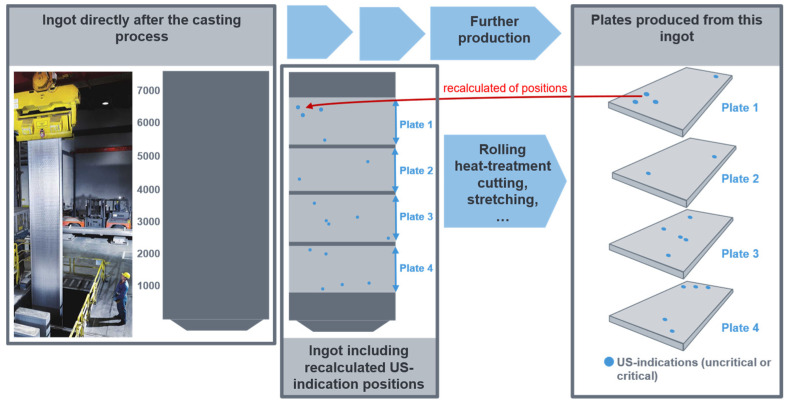
Recalculation of the US indications from the plates to the original ingot.

During the US test, the precise position (thickness, width, and length coordinates) of the defects and their dimensions are identified. These coordinates are then calculated, in a first step, back to the ingot, as illustrated in Figure 3. On the left side of the image, you can see the ingot immediately after casting (first grey box). The photograph illustrates the extraction of the ingot from the casting pit by an employee immediately after casting. To the right, a schematic representation of the ingot is displayed. At this stage of production, it is not yet known whether the ingot contains non-metallic inclusions or from which parts of the ingot the plates will later be produced.

The cast ingot is then subjected to a series of subsequent production processes, including rolling, heat treatment, cutting, and stretching, to create the final plates. The finished plates cut from this ingot can be viewed in the graphic located in the right-hand box.

The subsequent step in the process is ultrasonic testing. Ultrasonic testing is a method of identifying non-metallic inclusions and determining their exact coordinates within the plate. This includes determining the position in relation to thickness, width, and length, as well as the size of the defect. The detected US indications can be seen on the individual plates in the right-hand box. These can be both critical and non-critical defects or imperfections, which can be attributed to non-metallic inclusions. The coordinates are then ‘calculated back’ to the original rolled ingot from which the plates were rolled. This involves the consideration of the entire process chain, including the steps of homogenisation, rolling and stretching of the ingot, as well as the reconstruction of the defect position in the starting material. The recalculated defect and plate positions in the original ingot are shown schematically in the middle box. The dark grey areas represent sawing waste that was produced during the manufacturing process of the plates. For more details see [2,10].

It is imperative to implement a seamless digital recording system for all sawing, milling, and cutting operations throughout the production process. This will facilitate the calculation of the defect position back to the casting ingot. Even temperature differences in the material during production must be taken into account in order to account for density variations in the material. Any inconsistencies in the data acquisition process will result in significant errors in the calculated coordinates on the ingot, due to the considerable dimensional change.

### 2.2. Industrial Challenge and Specific Implementation Scenario

#### 2.2.1. General Challenge

Multi-stage production processes are established in a large number of industrial sectors. A recurrent phenomenon is the occurrence of errors in the production process, which are only identified at the end of the process—for example, during the final quality check [3]. This complicates the identification of causal relationships between specific production parameters and the detected defect, as relevant data must be linked across several process steps in order to precisely determine the error position in the product at the moment of occurrence. This, in turn, is required to make the analysis of the associated process parameters possible. Furthermore, intermediate steps in production between the occurrence and detection of the error can influence the final error pattern [3]. As demonstrated in the example provided, intermediate steps have the capacity to influence the size of the error.

The determination of a valid target variable and the development of robust analysis models are therefore challenging tasks. In many cases, the original process step that led to the error is known, which simplifies the analysis—but the analysis remains challenging, nonetheless. One such example is that of non-metallic inclusions during the casting process (see Section 2.1) [2,6,10]

First, it is necessary to identify the intermediate production steps that could influence the occurrence or detection of defects and determine the nature of this influence. In most industries, there is a wide range of product variants, which can manifest as differences in size, shape, material, or surface. Furthermore, there are often various facilities for producing the products in question. Intermediate production steps that could influence defect detection often become apparent when analyzing reject rates or error characteristics between different variants [3,6].

It is usually not sufficient to focus on specific variants in analyses because the production volumes for individual variants are often relatively low, particularly for high-quality specialty products. Therefore, the development of methods for transforming data from different variants to ensure comparability and enable cross-variant analysis is essential. It should be noted that data-driven statistical methods only lead to satisfactory results if there is a sufficiently large sample size of variants. In the following section, a method using FEM simulations will be presented, which offers advantages over statistical methods, particularly for smaller sample sizes.

#### 2.2.2. Specific Application—Dependence of the Reject Rate on the Final Plate Thickness

AMAG’s product range is comprehensive and includes a wide variety of plate formats, alloys, and ingot formats. In addition, a considerable number of different systems are available for production. In the following sections, one specific casting system, alloy, and ingot format will be the focus of the discussion. However, the method described can be applied to other casting systems, alloys, and ingot formats.

As already mentioned, the aim in this application example is to use statistical methods to find possible influencing variables on the formation of non-metallic inclusions during casting (measured via ultrasonic tests at the final plates). One of the biggest challenges is the strong dependence of the size of ultrasonic indications, measured on the final plates, on the rolling process, which becomes visible in the dependence on the final plate format.

**Figure 4:**
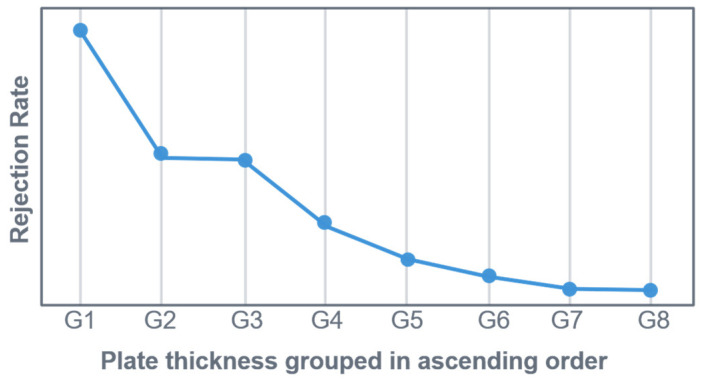
Dependence of the reject rate on the final plate thickness (one specific casting system, alloy, and ingot format).

Figure 4 illustrates the rejection rate, based on US indications, for the selected data set (one particular system, alloy, and ingot format).

To create a clear and concise graphical representation in Figure 4, the plate thickness was divided into nine equal groups (G1 to G8). Group G1 contains the thinnest plates, and the thickness increases with each successive group (G2, G3, etc.).

The x-axis represents the final plate thickness group (G1 to G9). The y-axis shows the mean rejection rate (based on US indications) for each group. Even when considering only one specific alloy, one casting format, and one casting plant, it is evident that the rejection rate is significantly influenced by the final plate thickness of the product. As the number and format of plates produced from the ingot are unknown directly after casting, there should be no correlation between the rejection rate (based on US indications) and plate thickness. This is because the cause of the US indications originates from the casting process. Therefore, it is evident that the manufacturing processes for specific plate thickness formats directly impact the rejection rate. This demonstrates that there is no comparability with regard to US scrap between products with the same alloy and ingot format from the same casting plant, but with different final plate thicknesses. It can also be shown that there are huge differences in the scrap rate between different alloys and plants. Nevertheless, this issue will not be addressed in any further detail in this article. This article only concentrates on only one specific casting plant, one alloy, and one ingot format.

The aspect of the rejection rate’s high dependence on the final plate thickness raises the question of what implications this has for statistical analyses or machine learning approaches. Due to the fact that the products examined are high-quality specialty products for the aerospace industry, production volumes are relatively low compared to other products. Consequently, it is not feasible to concentrate on individual final plate formats in the analysis to overcome this issue. In principle, the aim is to create the most exhaustive database possible, in which the products can be compared. The utilization of data transformation methods serves this purpose, with a detailed description provided below.

However, it is first necessary to identify the cause of this behavior. It is already known that the dependence on the final plate format is generated by several different factors. One cause is, for example, the dependence of the US-untested area in thickness direction on the final plate thickness. A detailed description of this behavior can be found in [10], which also presents a solution approach for accounting for this effect using statistical methods when calculating a target variable. However, this aspect is not considered further here. In the following, only the thickness range, which is always US-tested for all plate formats, is considered in order to be able to ignore this aspect and focus on other issues. For further consideration of this aspect, the explanations in [10] can then be followed.

Another cause is the presence of varying rolling forces, dependent on the final plate format, when the cast ingots are processed into plates. This topic is discussed in more detail below, and a methodology for calculating the target variable is presented.

**Figure 5:**
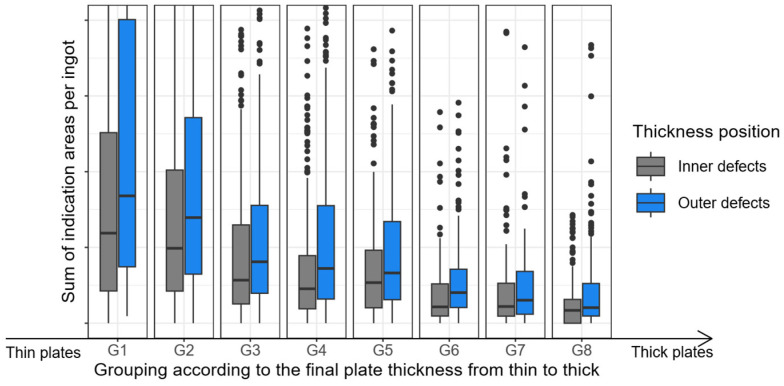
Effects of plate thickness on indication size (points indicate occasional outliers outside the whisker limits).

Figure 5 shows the different plate thickness groups G1, …, G8 on the x-axis. These labels are used to categorize plates according to their manufactured thickness. G1 stands for the thinnest plates, while G8 stands for the thickest. The groups are sorted in ascending order, i.e., $i=1,\ldots ,7:\;\forall \;g\in G_{i}\;\wedge \forall \;h\in G_{i+1}:\;g<h$.

Two box plots are shown for each plate thickness group. It is important to note at this point that only plates of a single thickness can be produced from a single ingot. Defects in ingots are classified according to their position in the produced plate in thickness direction as either ‘Centre’ (in the middle of the ingot in thickness direction) or ‘Outer’ (at the top and bottom of the ingot in thickness direction). The defect areas (US indication areas) in each ingot (or, more precisely, in all plates produced from the respective ingot) are then grouped according to ‘central’ and ‘outer’ and summed up. This provides the total defect area for each ingot, categorized as ‘central’ and ‘outer’, as illustrated in the boxplots.

The results demonstrate that as the plate thickness increases, the sum of defects area per ingot decreases, both for ‘central’ and ‘outer’ defects. The reason for this behavior is that the defect size decreases with increasing plate thickness due to different rolling pressures. For precise details, please refer to [10]. Furthermore, it has been determined that an increase in the final plate thickness results in a corresponding decrease in the number of indications that fall below the detection limit for ultrasonic testing [10]. To summarize, Figure 5 demonstrates that the thickness of the finished plate has a substantial impact on the size of the indications detected during ultrasonic testing.

As a direct consequence of this observation, it is essential to consider differences in rolling pressures when calculating the target variable. This ensures that the target variable accurately reflects casting-related non-metallic influences, regardless of any rolling effects.

The target variable, which is independent of rolling parameters, is a key requirement for the statistical analysis of potential influencing factors on casting quality and machine learning algorithms. It also provides foundry technologists with a valuable tool for evaluating the current casting quality.

### 2.3. Solution Approach and Use Case Implementation

#### 2.3.1. General Approach

The present study aims to address the question of how adequate target values can be defined in multi-stage industrial processes. Multi-stage industrial processes are to be found in almost all areas of industrial manufacturing and processing [3]. These processes are characterized by a series of interconnected steps, frequently involving a degree of interdependence, with the objective of yielding a final product from the initial raw materials. In the following, the challenges and potential solutions are discussed, as this issue manifests itself in a variety of industrial areas.

The utilization of the pure reject rate as a target value necessitates critical examination. In any case, a thorough examination is necessary to ascertain whether there are hidden correlations with process steps that are not taken into account in the data analysis [6]. In such cases, the utilization of the reject rate has the potential to introduce inconsistencies. There are a variety of approaches that may be adopted in order to enhance both the informativeness of the target variable and the robustness of the analysis. A comprehensive overview is provided in [6].

The presence of hidden correlations can pose a significant challenge as they reveal statistical relationships between the target variable and external variables that should not be regarded as influencing factors. An illustration of this is the thickness of the plates, which are not known at the time of casting. Nevertheless, as demonstrated previously, it exerts an influence on the rejection rate due to non-metallic indications. A range of statistical methods are available that are suitable for neutralizing such distortions. Common methods include grouping by confounding variables, variance stabilization, and transformations to a reference distribution [6,12,13]. These methodologies facilitate the establishment of a comparable target variable; however, they necessitate sufficiently large samples. In a significant number of industrial applications, the issue that arises is that a sufficiently large sample is not available for each production variant. The production of custom-made products and the high variety of variants to meet the requirements of a large number of potential customers results in a high heterogeneity of data, with individual groups often being underrepresented. In the cases previously mentioned, data-driven approaches frequently reach their limits. The subsequent section presents a case study exemplifying the effective integration of FEM simulations and data-driven statistical methodologies to adequately address the issue of limited sample sizes.

The fundamental question pertains to the systematic utilization of FEM simulations for the realistic and comparable calculation of defect patterns, such as non-metallic inclusions, in multi-stage processes—despite the complex interactions between the individual manufacturing steps. In many cases, the defect pattern (e.g., inclusions, distortion, cracks, dimensional deviations, etc.) that ultimately leads to scrap is caused by a single process step. However, the final extent of the defect is influenced by one or even several other process steps in combination [14]. For instance, it is possible that existing defects (inclusions, cracks, or shape deviations) may be further worsened under certain conditions. The presence of overlapping effects complicates the process of identifying the precise cause of the defect. The approach described aims to disaggregate these influences and achieve an objective, comparable assessment of the defect patterns.

In the initial stage, the manufacturing steps relevant to the defect pattern (e.g., forming, heat treatment, and machining) are simulated using an FEM model [15].

In order to identify the relevant manufacturing steps, a deep technical understanding is required. The objective of this approach is to obtain quantitative data regarding the extent to which this step impacts the subsequent defect pattern.

In the subsequent step, the defect pattern is ‘normalized’ using the FEM results. In this context, the term ‘normalization’ is to be understood as follows: the real defect pattern is corrected mathematically or statistically, considering the influencing factors whose effects are described by the FEM simulations.

The result shows a corrected error pattern that is independent of the influences of the individual ‘intermediate’ process steps. The corrected error values (and thus also the reject numbers) are adjusted for process conditions such as temperature, product format, etc. Using this method, it is possible to compare different production variants and evaluate, analyze, or predict reject rates for all products in a uniform manner. This results in an expansion of the database, which in turn enables the development of analyses and prediction models.

The method can be summarized as follows: each relevant production step is simulated individually to determine its individual relevance to the error pattern. Based on the simulation results, the influence of this contribution is mathematically extracted from the measured error patterns. This results in a standardized and comparable error measure, regardless of the extent to which, for example, forming or heat treatment were actually carried out. This approach allows error patterns to be calculated correctly and used for data analysis [6].

#### 2.3.2. Implementation in the Industrial Use Case

In the use case delineated in Section 2.2.2, the question arises about how the aforementioned dependency on rolling parameters should be taken into account when calculating the target variable. For this purpose, the degree of deformation was calculated for some individual final plate thicknesses using FEM simulations, depending on the final plate thickness. Further details are provided in Article [15]. It should be noted that direct experimental measurement of local deformation degree over the plate thickness is challenging and not practically feasible. For this reason, the present study uses FEM simulations to estimate the local deformation distribution. The validity of the FEM model is strongly supported by the close correlation between the simulation and experimental results for other key process parameters, such as roll separating forces, surface temperatures, and transversal thickness profiles of plates or strips and edge crack occurrence. The consistency of these results, obtained from multiple independent measurements, lends credibility to the model’s predictive capabilities, despite the absence of direct experimental validation of the local deformation [15,16,17].

This simulation for an individual final plate thicknesses was then extended to all possible plate thicknesses by calculating an estimate using regression for all possible plate thicknesses, resulting in a function for the forming factor as a curve over the ingot thickness, which depends on the final plate thickness. In more detail, firstly, a polynomial regression model is separately created for each final plate thickness in order to model the degree of deformation over the ingot thickness position. Subsequently, an additional polynomial regression model is formulated for each defect position, which delineates the degree of deformation as a function of the final plate thickness. The resulting models are then combined, and a function is defined that calculates the degree of deformation for any combination of final thickness and position in the ingot. The software R (version 4.4.2) was used to calculate the transformation function [18], with the following R packages [19,20] specifically utilized.

These statistical methods (linear regression) were used because an FEM simulation for each individual plate format would be very complex and resource intensive. By using regression models, the relationship between final thickness, defect position, and degree of deformation can be described mathematically and approximated. This allows the degree of deformation to be calculated efficiently for any combination of final thickness and position in the ingot without having to perform a separate FEM simulation for each variant (in total, an FEM simulation was only performed for seven plate thicknesses). This saves a considerable amount of time and computing effort, while still enabling a precise function for the forming factor.

**Figure 6:**
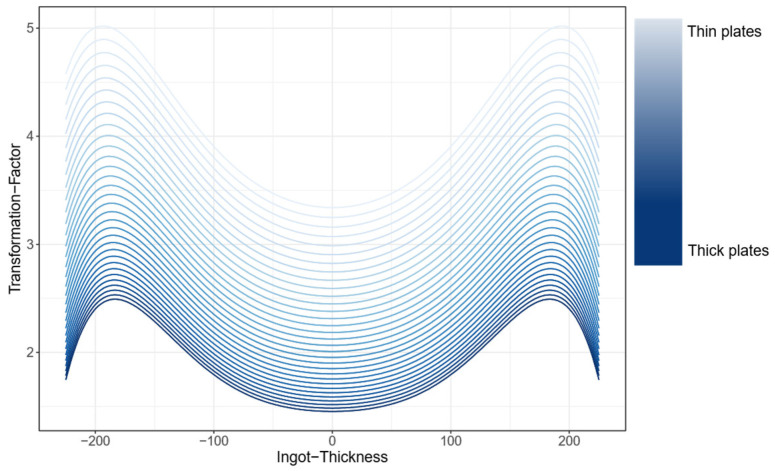
Transformation factor for different final plate thickness based on FEM simulations.

Figure 6 shows the curves of the forming factor as a function of the ingot thickness dependent on the final plate thickness [18,19,20].

As illustrated in Figure 6, the forming factor curves as a function of ingot thickness, contingent on the final plate thickness [18,19,20]. The x-axis indicates the position along the ingot thickness. Zero indicates the center point of the ingot in the thickness direction. The forming forces are symmetrical on both sides of the ingot in the thickness direction, and therefore the transformation factor must also be symmetrical.

Let us now consider the upper curve in the graph (light blue curve). This curve illustrates the transformation factor for defects in ingots that are rolled to the thinnest possible plate thickness. As can be seen in the graph, the defects at the edges of the ingot in the thickness direction, at approximately −190 mm and 190 mm, have the largest transformation factor. Defects in the center of the ingot at 0 mm have a smaller transformation factor.

The curve at the bottom of the graph (dark blue curve) shows the transformation factor for defects in ingots that are rolled to the thickest possible plate thickness. As a result, it can be concluded that the transformation factor is greater when the ingots are rolled into thinner plates.

From this function, which describes the transformation factor over the ingot thickness, an inverse function can now be calculated. This allows the defect size measured on the final plate (via US test) to be transformed, thus determining the actual defect size of the original defect in the ingot.

As previously stated, an increase in the final plate thickness will result in a greater number of US indications falling below the detection limit for ultrasonic testing. To take this into account, the detection limits are transformed from the final plate to the ingot in the same way. All indications that fall below the detection limit of the maximum plate thickness contained in the database are then removed, because these indications would not be detected if a plate with the maximum possible plate thickness had been produced from the ingot under consideration. This ensures comparability between the different plate thicknesses, and the target variable can be calculated based on the remaining indications.

**Figure 7:**
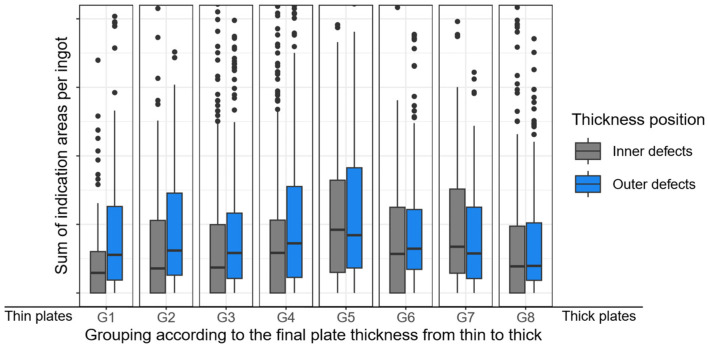
Transformed and reduced sum of indication areas per ingot for thickness range, which is always US-tested for all plate formats (points indicate occasional outliers outside the whisker limits).

Figure 7 now illustrates the transformed and reduced sum of indication areas per ingot by displaying box plots grouped by plate thickness. Similarly to Figure 5, Figure 7 illustrates plate thickness groups G1 to G8 on the x-axis, categorizing plates by manufactured thickness from thinnest (G1) to thickest (G8) in ascending order. Each group shows two box plots. Since only one plate thickness can be produced from a single ingot, defects are classified by their position in thickness direction as either ‘Centre’ or ‘Outer.’ The transformed and reduced sum of indication/defect areas from all plates of an ingot are shown for these two categories in the box plots.

It should be noted that Figure 5 demonstrated that as plate thickness increased, the sum of defect areas per ingot decreased, both for ‘central’ and ‘outer’ defects. As illustrated in Figure 7, this particular behavior is no longer observable when the transformed and reduced sum of indication areas are considered.

It is now evident that there is no longer a dependency on the final plate thickness. Consequently, the influence of deformation during rolling has been eliminated, and a comparability target variable between the different plate thicknesses is available for analysis.

## 3. Results—Analysis of Influencing Factors on the Target Variable and ML-Based Parameter Control

In the context of machine learning models and statistical data analysis for possible influences on product quality, the use of a comparable target variable is essential for the identification of significant and reliable patterns. Inconsistent definition or scaling of the target variable can lead to inaccuracy. This has a negative impact on performance and interpretability of results. Ensuring comparability of the target variable within the dataset used for machine learning models and statistical data analysis is therefore essential, as it enables the learning of relationships that are valid across the dataset. Consequently, the results become reproducible, and the model retains stability when applied to similar data sets. As part of data preparation, it is therefore essential to ensure a standardized definition of the target variables [6].

This section delineates the manner in which the target variable can be utilized to analyze the influencing factors in the casting process and how to control the identified influencing parameters using machine learning (ML) models. It also discusses the advantages of this method of calculating a target variable in comparison to other methods (for details see [6]). Firstly, the concluding definition of the target variable for the use case described in Section 2.1 is presented.

### 3.1. Use Case: Calculation of a Quality Measure per Batch

The target variable is calculated with consideration for the entire process chain, from the casting of the ingots to the final ultrasonic testing of the plates. The following aspects were considered:

1. Influence of defect position and final plate thickness: The position of the defects in thickness direction and the final plate thickness have been demonstrated to influence the defect size. The modelling was conducted utilizing FEM simulations and statistical methodologies. This modelling was integrated into the recalculation of the defect size from the final plates to the original ingot as described in Section 2.3.2.
2. Estimation of the defect area in the US-untested area: It should be noted that certain areas of the plates, due to technical constraints, could not be included in the testing process. Depending on the plate thickness, this affects 4% to 24% of the ingot (see [10]). For the US-untested areas, the defect area was estimated using an ML model—depending on the plate thickness and the defects found in the tested areas. Additional information is presented in [10].
3. Target variable per ingot and batch: The calculated defect areas result in a target variable per ingot (sum of defect areas per US-tested ingot weight). The target variable per batch is calculated as the median of the ingot target variables—excluding outliers that were previously identified and excluded by Monte Carlo simulations. Further details are available in [6].
4. Section-by-section quality assessment: The target variable was also calculated for individual sections of the ingot in order to detect changes in quality during the casting process. See [2] for details.

### 3.2. Analyis of Influencing Parameters

#### 3.2.1. Use Case: Analysis Result

It is well established that temperatures of all kinds frequently assume a pivotal role in production, particularly during the casting process. During the casting process, hundreds of different signals, including numerous temperature signals, are recorded. Feature selection methods were used to evaluate the relevance of this data with regard to the defined target variable [2]. This made it possible to identify the most meaningful signals and prioritize them for further analysis.

The subsequent section will focus on a specific signal. For reasons of confidentiality, the exact name of the signal cannot be disclosed. Consequently, the signal will be designated Signal ‘TS’ in the ensuing discussion.

**Figure 8:**
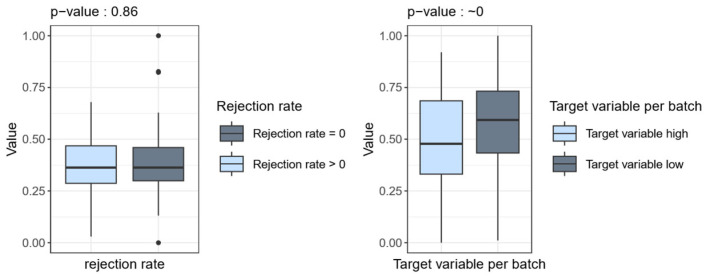
Boxplot of normalized Parameter $P_{TS}$ form Signal ‘TS’ for good- and poor-quality products classified according to rejection rate (left side) and low/high quality measure per batch (right side); points indicate occasional outliers outside the whisker limits.

The Signal ‘TS’ is recorded throughout the entire casting process, which lasts approximately two hours. This continuous recording enables a detailed analysis of the signal curves over the entire duration of the process. Data-driven investigations have identified a specific parameter within this signal ‘TS’ that has a significant influence on the final product quality [6,21]. This correlation is clearly evident in the calculated quality measure per batch, which serves as the target variable, as shown in Figure 8. The evaluation proves that a certain pattern in the Signal ‘TS’ can be attributed to quality deviations. This pattern is called Parameter $P_{TS}$ (Parameter of TS) in the following.

Figure 8 shows a comparison of the Parameter $P_{TS}$ between good- and poor-quality products using box plots (for more details see [6,21]). In the left-hand box in Figure 8, the rejection rate is displayed. A comparison is made between two quality groups: ‘Rejection rate = 0’ (illustrated in grey) and ‘Rejection rate > 0’ (illustrated in blue). Ingots from which only plates not rejected at the final stage of production were produced are compared with ingots from which at least one plate that was rejected was produced. The values of the signal “TS” are displayed on the y-axis. For each of the two groups, ‘Rejection rate = 0’ and ‘Rejection rate > 0’, the values of the signal ‘TS’ are displayed as a box plot. No significant difference between the two quality groups can be seen here. Furthermore, a non-parametric test was conducted utilizing the R package npmv [22], resulting in the calculation of the *p*-value. The null hypothesis, that there is no significant difference between the two quality groups, was confirmed by the high *p*-value of 0.86 [22].

A non-parametric test was used because the distribution of the data does not meet the requirements for classical (parametric) tests, such as normal distribution or equal variances. Non-parametric tests are more robust against outliers and work reliably even with small or skewed samples. They facilitate valid hypothesis testing even when the data structure is complex or the groups are of different sizes, as is often the case in industrial environments.

On the right-hand side of the figure, the target variable, which has been calculated as outlined in the preceding sections, is displayed. The target variable was divided into two groups. The group designated ‘Target variable high’ (Target variable > median(Target variable)) comprises all ‘bad’ ingots, and the group ‘Target variable low’ (Target variable < median(Target variable)) contains all ‘good’ ingots. ‘Bad’ ingots are characterized by a comparatively large number or size of defects, based on a comparable target value calculated methodically in the previous section, and are therefore considered to be of lower quality. The same principle applies to ‘good’ ingots. For each of the two, the values of the signal “TS” are once more displayed as a box plot, which indicates a significant difference with a *p*-value close to zero (utilizing the R package npmv [22]).

This finding, shown in Figure 8, underscores the conclusion that the correlation between the Parameter $P_{TS}$ and product quality cannot be adequately represented by the rejection rate. Only by using the calculated quality measure per batch does this correlation become evident. The reason for this is that the rejection rate still contains ‘hidden correlations’ with the final plate thickness, which distort the analysis result [6].

#### 3.2.2. General Approach

In this section, the rationale behind the meticulous definition of the target variable is examined. Section 3.2.1 provides answers to these questions using an example for illustration.

The first step in a data-driven analysis is to identify the relevant factors influencing the calculation of the target variable. It is of crucial importance here to understand and correct process-related influences, also known as ‘hidden correlations’ (see Section 2.2.1). The methodology for calculating the target variable described in Section 2.3 enables the identification of significant factors influencing product quality, which would not be evident when using alternative approaches, as shown in the example in Section 3.2.1.

In addition to the method presented in Section 2.3 for calculating a valuable target value using FEM simulations, there are numerous other approaches to data transformation that aim to correct hidden correlations. In [6], several methods are presented and applied to the example presented in Section 3.2.1. It was found that the transformations can improve the target variable. However, the calculation of the quality measure per batch described in Section 3.1 delivers the best results. Although it is the most complex method, it is also the most precise, especially for small sample sizes. The other transformation methods described in [6], on the other hand, only work reliably with large sample sizes and are therefore limited in their application, especially in industrial environments.

The relevance of this approach is evident for the following reason: the targeted analysis and modelling of quality-relevant parameters (as described in the following section) provides valuable insights that contribute to the optimization of the production processes, particularly with regard to reducing scrap and ensuring consistent product quality.

### 3.3. Application—Controlling Influencing Parameters Using ML Models

#### 3.3.1. General Approach

Machine learning models are becoming increasingly relevant in industrial manufacturing. The aim is to ensure product quality, reduce the rejection rate and use resources more efficiently. A key advantage of ML models is their ability to control critical parameters in a targeted manner. The full potential of this data-driven approach is particularly evident in automated and dynamic production environments [3,14,21]. As previously stated in the introduction, the three articles [7,8,9] provide a comprehensive overview of the most recent literature in this scientific field.

A concrete example is the parameter $P_{TS}$, whose influence on product quality can be proven to be significant, as shown in the previous Section 3.2. Deviations from the optimal range usually lead to quality deviations and increased scrap. To solve this problem, a machine learning model is being developed with the aim of controlling the parameter in a targeted manner. This method is designed to stabilize the signal value and adapt it to the given conditions. The model uses historical process data and relevant influencing factors to make accurate predictions.

Essentially, two issues can be identified that need to be examined more closely. The procedure described below can be applied to general industrial processes in which products are manufactured in batches, the conditions prior to the start of the process differ from those during the production process and signal values are continuously recorded throughout the entire production process.

The first question relates to predicting a signal value based on information that is available at the start of the process (example: start of casting). The aim is to use suitable modelling to enable the most accurate possible estimation of the expected signal value at the start of the production process, taking into account external factors (in the example: factors such as flow times in the melting channel system from the melting furnace to the mold, times in melt cleaning systems or ambient conditions).

##### Question 1: Predicting the Signal Value at the Start of the Process

- Data basis for the model:
  The utilization of historical process data for batches that have already been produced in recent years is a key component. In this context, it is essential to ensure that the production process has not undergone significant changes during the period under consideration. Furthermore, specific external factors at the time of process initiation (e.g., ambient temperature, raw material properties) are utilized.
- Model:
  The forecast is derived from the observed start conditions and historical patterns. The prediction is made for a single value per batch or process start. In such circumstances, static models such as regression or classification methods can be employed. Consequently, a conventional time series analysis is not required in this instance.
- Data preparation effort:
  Preliminary data preparation and validation is a necessity. In comparison with question 2, the computing time and streaming requirements are lower.
- Model evaluation:
  The accuracy of the signal value prediction at the initiation of the process and the stability under various start conditions are of paramount importance in this context. In the process of feature selection, it is firstly imperative to allocate particular attention to the identification of potential parameters that can be utilized to regulate the signal value at the start of the process. It is important to note that not all parameters can be controlled in a targeted manner (e.g., waiting times between two events). Consequently, it is crucially important in this context to keep these parameters as stable as possible across different start conditions.

The second question addresses continuous prediction during the ongoing production process (for example [3,23]). The aim of the model is to respond dynamically to changes during the production process and in the final step to intervene in a controlling manner if necessary.

##### Question 2: Real-Time Prediction During the Process

- Data basis for the model:
  Firstly, the model utilizes historical process data for batches that have been produced in recent years. Secondly, continuous sensor data is used during ongoing operation. This constitutes a fundamental distinction from question 1. At this point, it is important to note that external factors may be subject to change (e.g., fluctuations in metal flow, external conditions, etc.) during the production process.
- Model:
  In this model, chronologically sequential process and sensor data play a pivotal role. It is imperative that alterations over time, such as trends or deviations, are detected and incorporated in real time. For this purpose, time series models can be utilized.
- Data preparation effort:
  In contrast to question 1, continuous availability of data throughout the entire forecast period is required (e.g., IoT data). Furthermore, it is imperative to undertake real-time verification of data quality and model performance.
- Model evaluation:
  In addition to the challenges outlined in question 1, there is also the issue of forecast quality under time pressure, as well as the response speed of the model. This is undoubtedly also pertinent to question 1, but is likely to be an even more significant challenge in question 2, given that parameter settings frequently only impact the parameter to be controlled following a specified time delay. This is described in more detail in Section 3.3.3.

In addition, it is necessary to discuss how the prediction models can be used productively in the IoT environment. Further challenges are outlined in Section 3.3.3.

#### 3.3.2. Use Case

The implementation of these ML models involves the evaluation of historical data, with consideration given to data from batches since 2018 (comprising various alloys). It should be noted that the production specifications vary depending on the alloy.

With regard to question 1, an initial prediction model was developed based on a random forest regressor. The aim was to improve existing forecasts and create a robust basis for further analysis.

**Figure 9:**
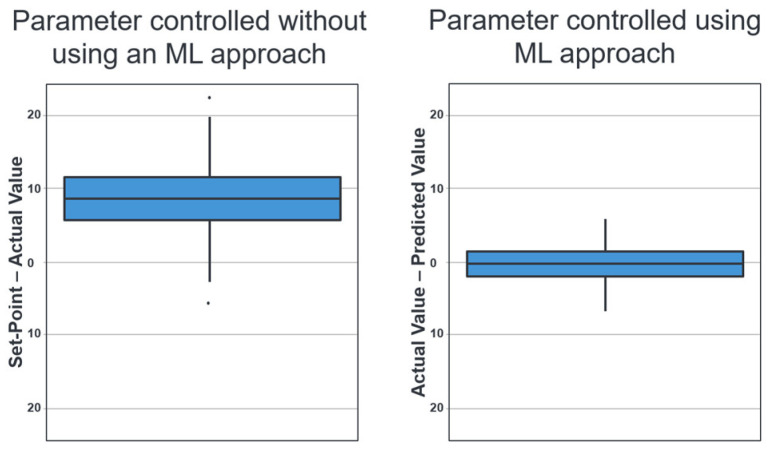
(**Left**): Parameter controlled to date without using an ML approach. (**Right**): Parameter controlled using an ML approach (points indicate occasional outliers outside the whisker limits).

Figure 9 on the left side shows how the parameter has been controlled to date without using an ML approach. The y-axis shows for each produced batch the difference between the input parameter (set point), which is specified as the target value, and the actual value. It is very clear that the actual value is almost always below the target value.

Figure 9 (right side) shows the first prototype of an ML approach (random forest) for better control of the parameter [18,19,20,24]. The R packages [18,19,20] were used for preparing and graphically representing the data. A random forest regression model was trained for modelling using the R function randomForest() [24]. The sample size excluding missing/invalid values is approximately 1200 batches. For reasons of confidentiality, the exact input parameters (features) cannot be disclosed. However, these are essentially process and production parameters that are relevant (e.g., measured temperatures in the casting channel, heating power, etc.) and have a direct influence on the target variable (parameter P_TS). The features were selected using standard methods, such as evaluating feature importance, to ensure robust prediction quality.

The model was built using a training dataset, which comprises 80% of the available data as standard. It consists of 500 decision trees in total (ntree = 500), in order to provide a robust estimate of the target variable and reduce model variance. The remaining 20% of the data was used to create a test set with which to evaluate the quality of the model (described below). A third of the available predictors were randomly selected for each split with a standard setting (mtry = ⌊p/3⌋, where p is the number of input variables). The trees were built using bootstrap samples with the same number of observations as the training dataset (sampling with replacement) and were trained without any explicit limit on the maximum number of nodes (maxnodes = NULL), up to a minimum node size of five observations (nodesize = 5). Additionally, variable importance calculation was activated (importance = TRUE), enabling the impact of individual input variables on model quality to be quantified using mean decrease in accuracy.

The graph on the right-hand side of Figure 9 shows the difference between the actual signal value of each batch in the test dataset and the value predicted by the model. It can be seen that the prediction deviates much less from the specified value and that there is no longer any overestimation or underestimation.

The following key figures were used to evaluate the model quality [18]: the root mean squared error (RMSE = 2.56) is a quantitative key figure that describes the average deviation of the forecasts from the actual value in the original units of the target variable. A root mean square error (RMSE) of 2.56 therefore means that the forecasts fluctuate by this amount on average.

According to the model R^2^ = 0.65, the model explains a variance of around 65% in the target variable. It was found that it is possible to map a significant proportion of the dispersion in the data. However, there is still room for improvement.

The mean absolute error (MAE = 2.07) refers to the mean absolute deviation of the forecasts from the actual value. The calculated forecast deviation of 2.07 is lower than that of the RMSE of 3.01, which indicates that the ‘typical’ errors are closer to the larger errors than previously assumed.

The model represents a significant improvement over the previous control system. With R^2^ = 0.65 and an MAE of 2.07, the developed model already delivers very solid prediction quality for a first prototype and provides a robust basis for further development.

The random forest model was chosen as the prototype because it provides a robust baseline for regression tasks involving complex, potentially non-linear relationships. The method achieves high stability against noise and outliers by combining many decision trees with random sampling and feature selection. Compared to other non-linear models, it also requires minimal parameterisation. It delivers good predictive quality without the need for complex hyperparameter tuning, and it enables an initial assessment of the relevant input variables via a feature importance evaluation. These features make the method suitable as a reference model or prototype for further model comparisons. This is explained in more detail in the following and in Section 3.3.3, particularly with regard to the importance of model interpretability.

After an initial model comparison (random forest, XGBoost, linear models [18,24,25,26]) was carried out and no optimization could be achieved compared to the current random forest model, the focus of further work will now be on expanding and improving the features.

Therefore, the next step is feature engineering. The aim is to increase the information in the input data in order to provide the model with additional parameters. The focus should be on the use of domain knowledge. The derivation of variables that reflect specific process- or material knowledge is an essential aspect of this step.

After implementing the feature expansion, a comprehensive validation of the model quality is planned, including cross-validation and residual analysis.

This further work aims to use an extended feature base to evaluate whether it is possible to optimize the existing random forest model. In this context, hyperparameter tuning will be used, which includes various methods such as grid search, random search, and Bayesian optimization [27]. A recent review of alternative model approaches will also be conducted.

#### 3.3.3. Further Work and Challenges in General

In addition to further model development, operational feasibility must also be examined. This includes, in particular, the availability and data quality of the input parameters for the model at the time the model makes a forecast. Incorrect data can lead to wrong predictions. It is therefore imperative to verify the data for plausibility and consistency, and to correct any discrepancies [28]. Furthermore, a comparison of the model types in terms of their performance and interpretability is necessary. The decision in favor of an explainable model depends on the priorities. An explainable model is preferred when safety and traceability are more important than marginal performance gains.

In industrial applications, explainable models are preferred because transparency and traceability are crucial for process reliability and compliance. The use of black-box models is only justified in rare cases by minimal performance gains, particularly due to security and acceptance issues. In this case, risks must be compensated for by additional measures. In [28,29], for example, a perspective on this is presented in the automotive, chemical, and process industries.

Addressing question 2 poses even greater challenges in the field of data science, which will be discussed below.

In the second question, the problem could be described as time-variable, condition-dependent lags, and interval-based areas of effect. This is explained in the following, as it is found in various industrial areas. In [30], for example, a method is described for identifying delayed effects and cause-and-effect relationships in industrial sensor networks.

For each product or batch produced, several signals are recorded throughout the entire production period under specific product specifications. A major problem is that the signals can be shifted in time relative to each other, depending on the position of the sensors. The time delay (lag) is not constant. The dependence of the phenomenon manifests itself in two ways. On the one hand, there is a dependence on static factors, such as product specifications. On the other hand, there is a dependency on dynamic factors such as production speed or specific process fluctuations. The aim is to synchronize the signals in order to ensure comparability for predictions and further analysis.

**Figure 10:**
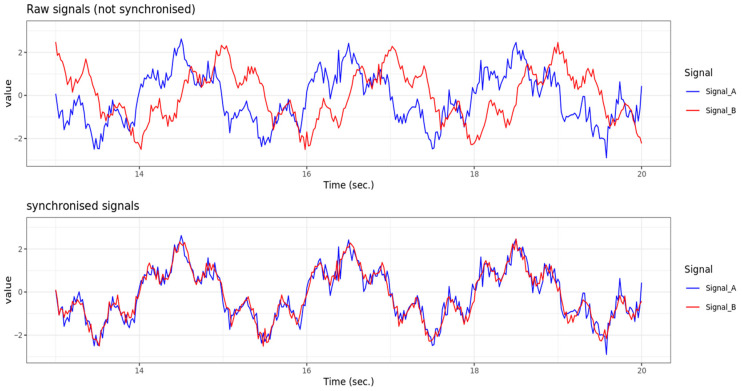
First plot: schematic example for two unsynchronized raw signals with time-shift of 0.5 s; second plot: synchronized signals.

Figure 10 shows a schematic example of synchronizing two raw signals. The upper diagram shows the two signals before synchronization, which have a time shift of 0.5 s. The x-axis represents time in seconds and the y-axis represents the signal values. This data was simulated using the R software [18]. The signal curves represent typical process data, such as that generated in the casting channel during the production of individual batches. Their temporal coordination is crucial for analysis. The lower diagram shows the same signals after synchronisation has been applied. This adjustment has completely eliminated the time shift, meaning the signals now run congruently. Synchronisation is necessary to enable correct feature extraction and reliable modelling. In addition, it must be taken into account that some signals are influenced not only by a single point in time, but also by behavior in a preceding time interval. Here is an example: the value of a signal at time x depends not only on another signal at time x–y, but also on the behavior of this signal in then interval (x–y1, x–y2). Therefore, the area of effect is defined by a time window, rather than a single point in time.

Future research will explore these issues in more detail. The aim is to develop a real-time forecasting model to predict the signal during production. With regard to the problem described above and the resulting questions, the following approaches to a solution are conceivable:

At the beginning of the research project, a baseline analysis should be carried out. For an initial assessment, this analysis may use methods such as cross-correlation and classic Dynamic Time Warping (DTW). These are time series analysis methods that enable the comparison of two time series [29]. The synchronized signals obtained in this way can then be used to train a prediction model for the target variables. The aim is to develop a model that considers time delays between signals and their areas of effect, based on product specifications and process factors (basics of time series analysis with R, see, for example, [31]).

#### 3.3.4. Summary and General Vision for the Future

A key advantage of machine learning models is their ability to control critical parameters in a targeted manner. This data-driven approach unleashes its full potential, particularly in automated and dynamic production environments.

A concrete example, which has been presented above, is the targeted control of a parameter that has been proven to have a significant influence on product quality (see Section 3.2). Deviations from the optimal range regularly lead to quality defects and increased scrap. To solve this problem, a machine learning model was developed that stabilizes the parameter and adapts it to the respective production conditions. It uses historical process data and relevant influencing factors to predict the value precisely.

As shown in Section 3.3.2, a prototype for predicting the start value of a relevant parameter has already been developed. This serves as the basis for the targeted control of the parameter at the start of the process. In the next steps, the model will be further developed to enable real-time prediction of the signal value throughout the entire casting process.

Such a model could provide plant operators with specific control recommendations to keep the signal value within a defined range. This would be a significant step towards intelligent process control. A future vision could also envisage such models being integrated directly into the plant control system and intervening automatically.

Implementing this vision would increase process reliability and product quality and significantly improve the efficiency and responsiveness of industrial plants. The use of ML models therefore represents a strategically sensible and technologically forward-looking approach for modern production facilities [28,29].

At this stage, the issue of collaboration between humans and ML models will be pivotal. The article [32] emphasizes the importance of collaboration between humans and robots. Humans are not viewed as replaceable components in a production process; rather, they are regarded as indispensable contributors to the value-added process. Robots (or machine learning models) are designed to strengthen human capabilities by fostering effective collaboration between employees and ML models, thereby enhancing productivity.

A comparable scenario is outlined in Article [33], which presents a method that can serve as a component of intelligent assistance systems in manufacturing. These systems will be capable of making autonomous decisions or providing recommendations in the future, such as recommendations for the optimal selection of tools, process monitoring, or the prevention of errors.

### 3.4. Financial Benefit

The targeted control and analysis of process parameters is a pivotal aspect of data-driven production optimization at AMAG. A substantial number of projects (one example is presented in Section 2.1 and Section 3.2, with further examples available in [21]) demonstrate that the utilization of data science, machine learning, and interactive data analysis tools, in particular, yields the following advantages for the company:

The systematic analysis of process data facilitated the identification of patterns indicative of quality deviations. As demonstrated in Section 3.2, the efficacy of utilizing data science to minimize scrap can be effectively illustrated. In this use case, a 70% reduction in scrap was achieved [21].

The example illustrates that the targeted control of identified quality-relevant parameters can lead to a reduction in scrap, optimized plant utilization, and shorter delivery times (see example in Section 3.3.2).

The synergy that has been cultivated among the production, technology, IT, data engineering, and analysis departments has been found to be remarkably efficacious.

Digitalization throughout the entire process chain facilitates the monitoring of machines and production processes [21]. The utilization of automated quality controls, leveraging artificial intelligence (AI) and predictive maintenance methodologies, is poised to play a pivotal role in the future by reducing machine downtime and ensuring product quality. Digital product tracking also facilitates complete traceability of errors [34].

These measures form the basis for innovation and competitiveness. The identification of quality-relevant parameters using data-driven approaches, and the subsequent specific control of these process parameters, is fundamental to the establishment of forward-looking, data-driven quality assurance. This is a pivotal factor in determining a company’s future competitiveness in the global market.

## 4. Discussion

This example describes an approach that can be used in various industrial processes, particularly in multi-stage production processes where complex interrelationships exist and traditional methods are ineffective.

First, a precise target variable must be defined. This target variable acts as a quality indicator and forms the basis for any data-driven optimization. It is crucial that this target variable accurately describes product quality and can be compared across different production conditions [6].

Multi-stage industrial processes consist of coordinated production steps that depend on each other, which can result in so-called ‘hidden connections’ [6]. These are correlations between final quality parameters and external factors that should not actually have any influence. One example from aluminum production is the final plate thickness of the rolled ingot, which remains unknown at the time of ingot casting yet still influences the rejection rate of casting related defects. This can interfere with correct analysis and require special methods to neutralize it. The results presented here indicate that combining data science methods with physics-based FEM simulations can significantly improve the definition of target variables for statistical analyses and ML models. Compared to classic, purely data-driven approaches, the presented method enables robust, comparable target variables, even with small sample sizes. This is particularly relevant in industrial practice, where many specialized products are manufactured in small quantities, and where classic statistical methods often reach their limits.

The second step is to identify and analyze the factors influencing the calculated target variable. This analysis can be performed using machine learning approaches, such as feature importance from ML models, or by applying domain knowledge, such as material and process properties. The aim of this step is to identify relevant production parameters to be specifically controlled, taking into account given boundary conditions such as initial conditions or dynamic process parameters such as flow velocity or temperature changes.

The results of the investigation confirm the working hypothesis that considering and correcting process-related influences (as in the example the rolling process) is essential for correct data analysis. Previous studies have highlighted the issue of ‘hidden correlations’ in production data [6]. The method developed in this context addresses this challenge by using FEM simulation and data transformations to generate a target variable that is independent of subsequent process steps. This enables optimized comparability of the target variable between different product variants.

A key finding of the study is that applying this method enables significant factors influencing product quality to be identified that would not be evident using alternative approaches. This can be illustrated by considering the casting signal ‘TS’ (example in Section 3.2.2), the influence of which on quality only becomes apparent through the transformation of the target variable using FEM simulation. The study emphasizes the importance of defining the target variable precisely for the successful application of statistical analyses and machine learning models in production contexts. Section 3.4 also describes the financial benefit that can be achieved by specifically controlling the signal ‘TS’, which has been identified as relevant to the quality of the final product.

The third step is where the real strength of machine learning comes into play. The results indicate that the Signal ‘TS’ (Example in Section 3.2.2) significantly influences the quality of the final product. This has far-reaching implications: being able to control critical process parameters specifically opens up new avenues for intelligent, data-driven process control. Even in the prototype phase, the ML model presented in this paper demonstrates considerable optimization compared to current control methods. In future, integrating such models into plant control systems could enable automatic, adaptive process optimization.

In addition, efficient data processing is essential. To this end, cloud storage as a scalable storage option (e.g., AWS, Azure) and advanced ETL (Extract–Transform–Load) processes must be implemented. These enable the extraction, transformation, and conversion of data from various sources into a standardized schema [35]. The use of high-performance computers or cloud-based platforms [36] is crucial for building an AI infrastructure.

Further steps also include the deployment of machine learning models within the domain of IoT environments, which gives rise to a number of technical and organizational challenges [28,37,38]. A pivotal factor in this regard pertains to the deployment location, namely whether it is situated on the edge, in close proximity to the machine, facilitating low-latency responses, or if it is hosted in the cloud, which offers augmented computational resources but may result in latency [37,38].

In order to guarantee accessibility for end-users, it is necessary to incorporate suitable services for model querying [38]. In addition, AI tools and analysis platforms are essential, such as Business Intelligence (BI) tools, machine learning platforms, and AI services [39,40,41].

Furthermore, the frequency of data transmission, latency, and required reaction times must be carefully analyzed, particularly in the context of real-time prediction scenarios. It is imperative that models deliver results within seconds; therefore, efficient buffering strategies must be implemented to prevent delays [38].

Model robustness is equally critical, requiring continuous monitoring of sensor data quality to detect anomalies such as outliers. It is imperative to meticulously monitor the performance of models in order to identify any deviations or discrepancies in predictions. Moreover, it is essential to incorporate mechanisms for automated or manual retraining of models in response to changes in process conditions. Finally, the model’s ability to handle measurement inaccuracies and data gaps is essential for ensuring reliability in real-world production settings (big data challenges in manufacturing see also, for example [3,14,28,29]).

Future work must address all these challenges in order to enable the use of the ML model, the first prototype of which was presented in this paper, in a production environment.

## 5. Conclusions

- In summary, the approach described in this work offers a significant advantage by using mathematical and statistical methods together with physics-based simulations to calculate a robust and comparable target variable. By neutralizing hidden correlations and process influences, it becomes possible to assess product quality reliably across all variants, even when only limited or imbalanced data is available. Machine learning models can then build on this foundation to identify and control the most critical quality parameters, leading to targeted process optimization and a measurable reduction in scrap. The study demonstrates the efficacy of this methodology not only for aluminum production, but also for other industries with complex processes and small datasets. Further research will concentrate on the following: extension of the method for calculating a target variable using FEM simulations to other alloys, formats, and production lines.
- Development of analysis methods for small, non-normally distributed datasets and imbalanced datasets.
- Development of real-time prediction models for process signals with time-variable, condition-dependent lags and interval-based areas of effect.
- Integration of real-time prediction models for productive use in IoT and cloud environments.

Overall, the integration of simulation, technological expertise, and data-driven analysis opens up new possibilities for robust and efficient production control in modern industry.

## Data Availability

The datasets presented in this article are not readily available because the data are part of an ongoing study and to allow for the commercialization of research findings.

## Abbreviations

The following abbreviations are used in this manuscript:

AI	Artificial Intelligence
AMAG	AMAG Austria Metall AG
AWS	Amazon Web Services
BI	Business Intelligence
DTW	Dynamic Time Warping
ETL	Extract, Transform, Load
FEM	Finite Element Method
MAE	Mean Absolute Error
ML	Machine Learning
RMSE	Root Mean Square Error
TS	Time Series
US	Ultrasonic
